# A Rare Genetic Defect of *MBL2* Increased the Risk for Progression of IgA Nephropathy

**DOI:** 10.3389/fimmu.2019.00537

**Published:** 2019-03-22

**Authors:** Yan Ouyang, Li Zhu, Manman Shi, Shuwen Yu, Yuanmeng Jin, Zhaohui Wang, Jun Ma, Meng Yang, Xiaoyan Zhang, Xiaoxia Pan, Hong Ren, Weiming Wang, Hong Zhang, Jingyuan Xie, Nan Chen

**Affiliations:** ^1^Department of Nephrology, Institute of Nephrology, Ruijin Hospital, Shanghai Jiao Tong University School of Medicine, Shanghai, China; ^2^Renal Division, Department of Medicine, Peking University First Hospital, Beijing, China

**Keywords:** complement lectin pathway, genetic variations, IgA nephropathy, disease progression, deregulated complement activation

## Abstract

The aim of this study was to investigate the association between lectin pathway-related genetic variations and progression in IgA nephropathy. Biopsy-proven IgAN patients with eGFR ≥15 ml/min/1.73 m^2^ at baseline and a minimum follow-up of 12-months were enrolled. A total of 1,007 patients and 121 healthy controls were enrolled from two Chinese renal centers. The discovery cohort consisted of 606 patients, and the validation cohort consisted of 401 patients. First, promoters, all exons and their boundary regions of *MBL2* and *FCN2* were sequenced in 50 patients, and then 37 variations were identified. Of these variations, 7 expression-associated variations were selected and genotyped in the whole discovery cohort. We found that rs1800450 in *MBL2* and rs7851696 in *FCN2* were associated with an increased risk for ESRD as well as serum MBL or L-ficolin levels. However, only rs1800450 was successively validated for its association with ESRD (HR, 15.91; 3.27–77.34; *P* = 0.001) in the fully adjusted model in the validation cohort. In addition, 2.7% of patients, and 2.5% of healthy controls carried rs1800450-AA. IgAN patients with rs1800450-AA lacked expression of MBL in both serum and renal tissue and had more severe tubulointerstitial damage. Furthermore, a combined effect of rs1800450-AA with a previously reported clinical risk score was observed in which patients with both a high clinical risk score (≥1%) and rs1800450-AA had a strikingly increased 10-years ESRD risk by 37.1-fold (7.17 to 192.13-fold). In summary, IgAN patients carrying *MBL2* rs1800450-AA have a high risk for renal function deterioration, probably due to inactivation of the complement MBL pathway.

## Introduction

Immunoglobulin A nephropathy (1) is an autoimmune disease characterized by mesangial deposits of the galactose-deficient IgA1-prominent immune complex. It is the most common primary glomerulonephritis worldwide (2–4). Up to 20–40% of IgAN patients eventually progress to end-stage renal disease (ESRD) within 20 years. Among them, the renal function of 5–10% patients rapidly deteriorates within 5-years (5). It is suggested that activation of the complement system plays a key role in the renal damage of IgAN, mainly through the lectin pathway and the alternative pathway (6–8).

Roos A and colleagues previously found that IgAN patients with renal deposition of MBL and L-ficolin had serious renal histological lesions (9). Guo et al. (10) found an increased risk for progression in IgAN patients with extremely low or high serum MBL levels; further analysis showed that patients with MBL deficiency had a worse prognosis than those without MBL deficiency. These studies indicated the important role of the lectin pathway in IgAN progression and that complement deficiency could contribute to renal damage. However, the underlying mechanism remains unknown.

As activators of the lectin pathway, MBL, and L-ficolin (11) can bind to microbes and apoptotic cells and subsequently form complexes with MBL-associated serine proteases 2 (MASP2) to trigger the lectin pathway, thereby leading to the elimination of those target agents (12). The coding genes of these complements might determine their serum levels and functional activity. The serum level of MBL has been reported to be associated with genetic variations in both promoter and coding regions of *MBL2* (NG_008196.1) (13–16). The functional activity of L-ficolin is also affected by *FCN2* (NG_011649.1) variations (17). Therefore, we hypothesized that the genetic background of these complement components affects activation of the complement lectin pathway and leads to the acceleration of IgAN progression.

To study the association of the genetic background of the lectin pathway with IgAN progression, we sequenced the two regulator genes *(MBL2 and FCN2)* of the MBL pathway and genotyped the candidate variations in two extended Chinese cohorts. Subsequent association analysis was performed between the candidate variants and disease progression by building a Cox proportional hazards regression model.

## Materials and Methods

### Participants and Demographic Characteristics

**Figure 1:**
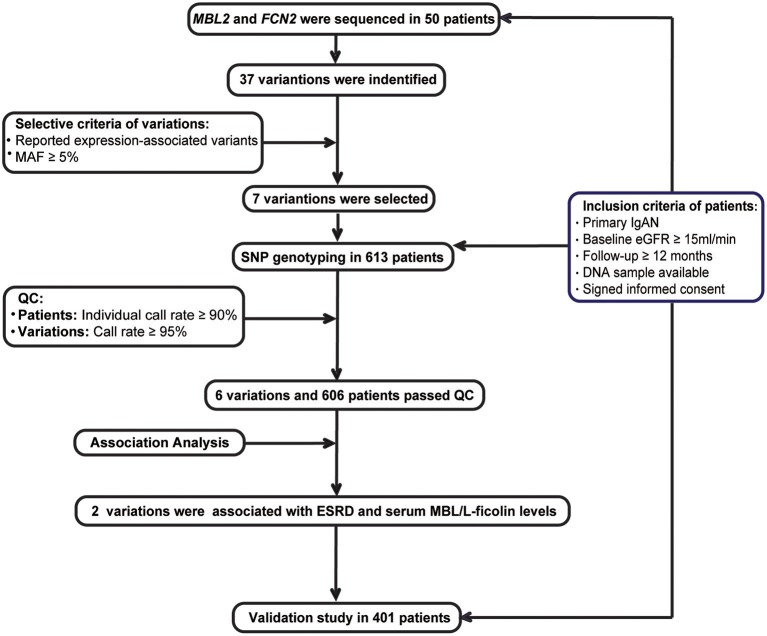
Study flow diagram.

Recruitment criteria for the IgAN patients included the following (Figure 1): (1) IgAN was defined by a renal biopsy demonstrating dominant IgA deposition in the mesangium of glomeruli by immunofluorescence microscopy; (2) secondary causes of Henoch-Schönlein purpura, systemic lupus erythematosus, or liver disease were excluded; (3) estimated glomerular filtration rate (eGFR) ≥15 mL/min/1.73 m^2^ at diagnosis; (4) minimum follow-up of 12-months; (5) DNA sample available; and (6) an informed consent was signed.

All patients were recruited from the two Chinese renal centers, namely, the Department of Nephrology, Ruijin Hospital, Shanghai Jiao Tong University School of Medicine (RJ), and the Renal Division, Peking University First Hospital (PKU). The discovery cohort consisted of patients from RJ who were diagnosed by renal biopsy before Jan 2014, while the validation cohort consisted of patients from RJ who were diagnosed after 2014 and all patients from PKU.

The clinical variables at the time of renal biopsy and during follow-up were recorded, including demographic indicators, such as gender and sex; physical examinations, such as systolic (SBP) and diastolic blood pressures (DBP); laboratory tests, such as serum creatinine (Scr) and 24 h protein excretion; and treatments, such as angiotensin-converting enzyme inhibitors (ACEI) and/or angiotensin receptor blockers (ARB) or glucocorticoids as previously described (18–20). eGFR was calculated using the Chronic Kidney Disease Epidemiology Collaboration (CKD-EPI) equation (21). The classification of chronic kidney disease (CKD) was based on the Kidney Disease Outcomes Quality Initiative (K/DOQI) practice guidelines (22): Stage 1: eGFR ≥90 ml/min/1.73 m^2^; Stage 2: eGFR 60–89 ml/min/1.73 m^2^; Stage 3: eGFR 30–59 ml/min/1.73 m^2^; Stage 4: eGFR 15–29 ml/min/1.73 m^2^; and Stage 5: eGFR < 15 ml/min/1.73 m^2^. Our previous reported CLIN-PATH equation (23) was used to calculate the risk for ESRD in individuals with IgAN: (1) 5-years risk for ESRD: 1–0.9725^exp{[−0.0323×Age−37.3)]−[0.0567×(eGFR−72.5)]+[0.6351×(M−0.39)+[0.7452×(T−0.53)]}^;(2) 10-year risk for ESRD: 1–0.9063^exp{[−0.0323×Age−37.3)][0.0567×(eGFR−72.5)]+[0.6351×(M−0.39)+[0.7452×(T−0.53)]}^.

Two experienced pathologists evaluated the renal tissue independently. The severity of the renal damage was scored according to the Oxford-MESTC classification (24). In detail, M1 was defined as a mesangial score >0.5. E or S was scored as absent (0) or present (1) endocapillary hypercellularity (E) or segmental glomerulosclerosis (S). T was scored according to the estimated percentage of tubular atrophy/interstitial fibrosis < 25% (T0), 26–50% (T1), or >50% (T2). C was scored by the estimated percentage of cellular/fibrocellular crescents as absent (C0), present in < 25% of glomeruli (C1), and present in >25% of glomeruli (C2). For further association analysis of baseline histological grades with variations, the tubulointerstitial damage was classified as non-severe (injury ratio ≤ 50%, T0 and T1) and severe (injury ratio >50%, T2). The crescents were also classified as non-severe (percentage ≤ 25%, C0 and C1) and severe (percentage >25%, C2).

### Outcomes and Definitions

The start of follow-up time was considered the date of renal biopsy. The predefined renal outcome of our study was ESRD (eGFR < 15 ml/min/1.73 m^2^ or the need for dialysis/renal transplantation). Patients were censored at the time of ESRD or loss to follow-up.

### Discovery Stage

Fifty cases were randomly chosen from the discovery cohort. The promoter, all exons and their boundary regions of the *MBL2* an *FCN2* genes were sequenced among these patients (Supplementary Table 1). Among all the detected variations, we selected common variations (MAF ≥ 5%), which were associated with their expression based on previous literatures for further genotyping. Based on the power calculation, we had at least 80% statistical power to detect a variant with a MAF of at least 5% with the assumption of an alpha of 0.05 based on the sample size of the discovery cohort (Supplementary Table 2). These candidate variations were then genotyped using the MassARRAY system in the whole discovery cohort. And then, the quality controls (QC) of patients and variations included a SNP call rate ≥95% and individual call rates ≥90%. Only variations and patients passed the QC were enrolled for the association analysis.

Next, associations of haplotypes or variations with ESRD were analyzed in the discovery cohort. Haplotypes were phased and estimated using PLINK v1.07. Haplotypes with frequencies < 0.1 were not enrolled in this analysis due to the insufficient statistical power. All the patients were divided into ESRD (progressed to ESRD within the follow-up period) and non-ESRD (did not progress to ESRD within the follow-up period) groups. The haplotype was selected as a reference due to its equal frequency between the progressive and non-progressive groups.

Moreover, the associations between candidate variations and ESRD were analyzed by using Cox proportional hazards regression model with parameters including sex, age, eGFR, systolic blood pressure, diastolic blood pressure, 24 h urinary protein excretion, and Oxford MESTC at the time of renal biopsy and treatment options (ACEI/ARB and glucocorticoids).

### Validation Stage

In the validation cohort composed of patients from RJ and PKU, the associations of the candidate variations with their serum levels or ESRD were validated. In addition, the associations between the candidate variations with their serum levels were further validated in healthy controls.

### Candidate Gene Sequencing and Analysis

Genomic DNA was extracted from peripheral blood using a GenElute blood genomic DNA kit (Sigma-Aldrich, USA). DNA samples with an A260/280 ratio ranging between 1.8 and 2.0 were included. Purified PCR products were directly sequenced using an ABI 3700 automated DNA sequencer (Perkin-Elmer Applied Biosystems, USA). Sequencing data were analyzed using Sequencher5.1 software and further confirmed based on the National Center for Biotechnology Information (NCBI) database and 1000 Genomes Project.

### SNP Genotyping and Analysis

SNP genotyping was performed using the Sequenom MassARRAY® iPLEX system (Sequenom, USA) according to the manufacturer's recommendations. The products were transferred onto 384-matrix spot chips following desalination, followed by analysis using the MALDI-TOF MS assay and genotyping using Sequenom Typer 4.0 software.

### Complement Activation Assay

The serum complement MBL and L-ficolin were measured in 282 IgAN patients (139 male: 143 female; 0.97: 1) and 60 normal controls (28 male: 32 female; 0.88:1) using the enzyme-linked immunosorbent assay (ELISA), according to the manufacturer's instructions. The ELISA kits were purchased from Hycult Biotech (Uden, The Netherlands). The color intensity was evaluated at 450 nm in an ELISA reader.

In 55 IgAN patients, renal depositions of MBL (Abcam, USA) were performed using an immunochemistry method described by Roos et al. (9). The deposition of L-ficolin (Biorbyt, United Kingdom) was assessed using a similar protocol.

### Statistical Analysis

The sample size of our study was performed using the Power and Sample Size Calculations software version 3.1.2. The haplotype test was phased with PLINK v1.07. Linkage disequilibrium was analyzed using Haploview 4.1. The normality of quantitative variables was assessed using the Kolmogorov-Smirnov test, and continuous data are expressed as the mean ± standard deviation (SD) or median (minimum-maximum). For normally distributed variables, groups were compared using the *t*-test or one-way analysis of variance. Groups with non-normally distributed variables were compared using the Mann-Whitney *U*-test or the Kruskal-Wallis test. Categorical data are expressed as frequencies or percentages (%) and were compared using a standard chi-squared test. Probabilities of cumulative renal survival curves were generated by the Kaplan-Meier method. Hazard ratios (HRs) with 95% confidence intervals (CIs) were calculated from Cox regression proportional hazards models. Linear regression and binary logistic regression were performed for correlation analysis. Correlation coefficients are presented as the standardized β (for continuous parameters) or odds ratios (for categorical parameters). Statistical analysis was performed using SPSS version 22.0 (SPSS Inc., USA). *P* < 0.05 were considered statistically significant. The random effect models of two cohorts combined were performed in the R statistical programming language using the “metafor” package (R, version 3.5.1; R Foundation for Statistical Computing, Vienna, Austria).

## Results

### Subject Characteristics

In total, 1007 IgAN patients and 121 healthy controls were enrolled in the study. The baseline demographic and clinical characteristics of the IgAN cohorts were summarized (Table 1). The discovery cohort was composed of 606 patients with IgAN (median follow-up, 40.7 months; range, 12–238 months). The validation cohort was composed of 401 patients with IgAN (median follow-up, 53.9 months; range, 12–211 months). The average age of the patients in the discovery cohort was 36.9 ± 12.2 years, including 307 males (51.0%) and 299 females. The average age of the patients in the validation cohort was 34.6 ± 11.6 years, including 199 males (49.6%) and 202 females. Compared with patients in the validation cohort, patients in the discovery cohort had significantly lower eGFRs (73.3 vs. 87.4 ml/min/1.73 m^2^), higher mean systolic (128.4 vs. 125.5 mmHg) and diastolic pressures (81.3 vs. 78.7 mmHg) and more patients with Oxford S1 (82.7 vs. 71.9%), reflecting a higher disease severity in the discovery than the validation cohort. However, ESRD occurred similarly in the two cohorts [83 of 606 (13.7%) vs. 59 of 401 (14.7%)] (Table 1). In addition, 121 healthy controls were recruited, consisting of 52 males (43%) and 69 females with a mean age of 51.1 ± 11.3 years.

**Table 1:** Baseline characteristics of All IgAN patients.

**Variables**	**Discovery cohort (*N* = 606)**	**Validation cohort (*N* = 401)**	***P*-value**
Follow-up, months	40.7 (12–238)	53.9 (12–211)	–
Developed ESRD	83 (13.7)	59 (14.7)	0.7
Dge at biopsy, years	36.9 ± 12.2	34.6 ± 11.6	0.003
Dale gender	307 (50.7)	199 (49.6)	0.7
DGFR, mL/min/1.73 m^2^	73.3 (15.4–163.9)	87.4 (15.7–140.2)	<0.001
**CKD CLASSIFICATION^a^**			
DKD stage 1	205 (33.8)	187 (46.4)	-ref-
DKD stage 2	167 (27.6)	110 (27.4)	0.04
DKD stage 3	174 (28.7)	78 (19.5)	<0.001
DKD stage 4	60 (9.9)	26 (6.5)	0.003
DBP, mmHg	128.4 ± 17.2	125.5 ± 16.5	0.008
DBP, mmHg	81.3 ± 11.9	78.7 ± 12.2	0.001
Drine protein, g/d	1.3 (0.03–12.7)	1.2 (0.01–15.2)	0.8
**OXFORD CLASSIFICATION^b^**			
D1	178 (38.0)	146 (36.6)	0.7
D1	150 (32.0)	150 (37.6)	0.08
D1	388 (82.7)	287 (71.9)	<0.001
D1	113 (24.1)	89 (22.3)	0.5
D2	58 (12.4)	46 (11.5)	0.6
D1	187 (39.9)	190 (47.6)	0.025
D2	31 (6.6)	23 (5.8)	0.9
DCEI or ARB treatmentf^c^	410 (76.1)	366 (91.3)	<0.001
Dlucocorticoid treatment^c^	322 (59.7)	204 (50.9)	0.007

*BMI, body mass index; CKD, chronic kidney disease; eGFR, estimated glomerular filtration rate; SBP, systolic blood pressure; DBP, diastolic blood pressure; M, mesangial hypercellularity; E, endocapillary proliferation; S, segmental glomerulosclerosis; T, tubular atrophy and interstitial fibrosis; C, crescents; ACEI, angiotensin converting enzyme inhibitors; ARB, angiotensin II Receptor Blockers*.

a *Stage 1: eGFR ≥ 90 ml/min/1.73 m^2^; Stage 2: eGFR 60–89 ml/min/1.73 m^2^; Stage 3: eGFR 30–59 ml/min/1.73 m^2^; Stage 4: eGFR 15–29 ml/min/1.73 m^2^; Stage 5: eGFR < 15 ml/min/1.73 m^2^*.

b *868 patients had OXFORD-MESTC score*.

c *940 patients had treatment records*.

### Screening for Candidate Variations and Association Study in the Discovery Cohort

In the discovery cohort, after sequencing of the *MBL2* and *FCN2* genes in 50 patients, 37 variations were identified. Sixteen variants were found in *MBL2* (Supplementary Table 3A), including nine in the promoter region (rs11003125, rs7100749, rs11003124, rs7084554, rs36014597, rs10556764, rs7096206, rs189469831, and rs11003123), four in the intron region (rs4647964, rs1982266, rs930508, m.370–145A>T), one in the 5'UTR (rs7095891), and two in the coding region [rs1800450 (p. Gly54Asp) and rs930507 (p. Leu126)]. Twenty-one variants were identified in *FCN2* (Supplementary Table 3B), including six in the promoter region (rs3124952, rs3811143, rs3124953, rs373370111, rs3811140, and rs7865453), one in the 5'UTR (rs17514136), nine in the intron region (rs7032741, rs3124955, rs7024491, rs118122273, rs3128624, rs7037264, rs148404686, rs12684723, and rs4521835), and five in the coding region [rs4520243 (p. Arg74), rs12684476 (p. Gly117Ser), rs34789496 (p. His181), rs17549193 (p. Thr236Met), and rs7851696 (p. Ala258Ser)].

Then, based on the selective criteria of variations, seven expression-associated variations with MAF≥5% were selected for analysis, including 4 in *MBL2* (rs11003125, rs1800450, 7096206, and rs7095891) (25–27) and 3 in *FCN2* (rs3124952, rs17514136 and rs7851696) (17, 28), and further genotyped in the discovery cohort. According to the quality controls (QCs) of patients and variations, rs11003125 was removed due to its SNP call rate <95%, and 7 patients were removed for individual call rates <90%. Finally, 6 variations and 606 patients who passed the QC step were included for further association analysis.

Cox regression analysis showed that only 2 out of 6 variations were significantly associated with an increased risk for ESRD, as well as with serum levels of MBL or L-ficolin, including rs1800450 [AA vs. GG: HR, 4.47; 95% CI, 1.75–11.46; GA vs. GG: HR, 1.63; 95% CI, 1.03–2.59] in *MBL2* and rs7851696 (GT vs. GG: HR, 1.76; 95% CI, 1.12–2.77) in *FCN2* (Supplementary Table 4). We then performed haplotype analysis encompassing three variations (rs7096206, rs7095891, and rs1800450) in *MBL2* (Table 2). For the *MBL2* gene, we identified four common haplotypes. Haplotype-M1 (GCG) was selected as the reference due to its equal frequency between the ESRD and non-ESRD groups (53.5 vs. 57.4%). Additionally, only haplotype GCA (OR = 1.51, 95% CI, 1.01–2.27) was related to disease progression.

**Table 2:** Analysis of associations of *MBL2* haplotypes with ESRD in discovery cohort (*N* = 606).

**Haplotype**	**Markers**			**Frequency (%)**			**OR (95%CI)**
				**Total**	**ESRD**	**Non-ESRD**	
***MBL2***	**rs7096206**	**rs7095891**	**rs1800450**				
M1	G	C	G	56.8	53.5	57.4	-Reference-
M2	G	C	A	17.5	23.5	16.6	1.51 (1.01–2.27)^*^
M3	C	C	G	14.4	14.1	14.5	1.03 (0.64–1.67)
M4	G	T	G	11.2	8.8	11.6	0.82 (0.46–1.46)

* *P < 0.05. The whole patients were dived into ESRD and non-ESRD groups. Haplotype were phased and estimated by PLINK v1.07. Haplotype with frequencies < 0.1 were not enrolled for this analysis, due to the little power they would provide. We use the most common and equally distributed haplotype as reference, the odd ratio (OR) and 95%CI of other haplotypes were analyzed*.

**Figure 2:**
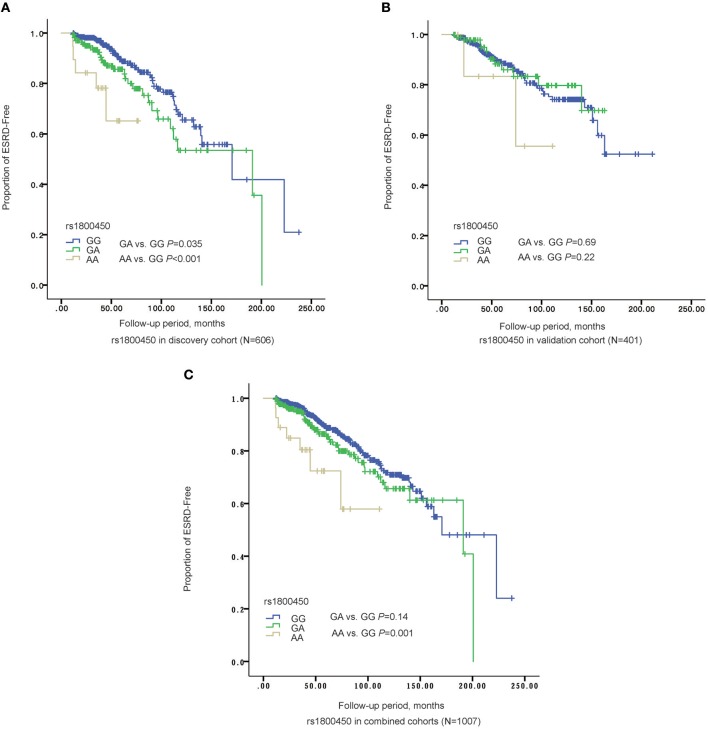
The Renal survival of different genotypes of rs1800450 in different cohorts. **(A)** In discovery stage (*N* = 606), AA group (mean ± *SD*: 60.0 ± 7.5 months, *P* = 0.035) had a significant shorter renal survival time than GA (mean ± SD: 140.0 ± 9.6 months, *P* < 0.001) and GG groups (mean ± *SD*: 161.6 ± 10.3 months); **(B)** In validation cohort (*N* = 401), AA group (mean ± SD: 85.9 ± 14.4 months, *P* = 0.22) had a significant shorter renal survival time than GA (mean ± S*D*: 139.1 ± 6.2 months, *P* = 0.69) and GG groups (mean ± *SD*: 161.4 ± 7.1 months); **(C)** In the combined cohorts (*N* = 1,007), AA group (mean ± *SD*: 82.4 ± 9.0 months, *P* = 0.14) had a significant shorter renal survival time than GA (mean ± *SD*: 150.5 ± 6.9 months, *P* = 0.001) and GG groups (mean ± *SD*: 167.7± 7.2 months).

Kaplan-Meier survival analysis revealed that either patients in the rs1800450-AA group (mean± SD: 60.0 ± 7.5 months, *P* < 0.001) or GA group (mean± SD: 140.0 ± 9.6 months, *P* = 0.035) had a significantly shorter renal survival time than patients in the GG group (mean± SD: 161.6 ± 10.3 months, Figure 2). The multivariate Cox analysis showed that only rs1800450 was independently associated with ESRD after adjustment for clinical and pathologic indicators (AA vs. GG: HR, 9.64; 95% CI, 2.40–38.71; *P* = 0.001). Moreover, each single A allele of rs1800450 could increase the risk for ESRD by 2.21-fold (1.24 to 3.94-fold) in the fully adjusted model. However, the association between rs7851696 and ESRD was only significant in the univariate Cox regression model [GT vs. GG: HR, 1.76; 95% CI, 1.12–2.77; *P* = 0.01], but it became insignificant in the fully adjusted model [GT vs. GG: HR, 1.57; 95% CI, 0.78–3.17; *P* = 0.21]. Similarly, each single T allele of rs7851696 increased the risk for ESRD by 1.53-fold (1.06 to 2.19-fold) by univariate Cox analysis, but they lost significance in the fully adjusted Cox regression model (Table 3).

**Table 3:** Cox regression analysis of ESRD risk and rs1800450 or rs7851696 in all patients.

**Group**	***N* (%)**	**ESRD (%)**	**MAF (%)**	**Model 1^b^**		**Model 2^c^**		**Model 3^d^**	
				**HR (95% CI)**	***P*-value**	**HR (95% CI)**	***P*-value**	**HR (95% CI)**	***P*-value**
**rs1800450 (G>A)**									
**Discovery cohort**	606		17.3	1.83 (1.26–2.66)	0.002	2.29 (1.44–3.62)	<0.001	2.21 (1.24–3.94)	0.007
GG	415 (68.5)	48 (11.6)		Reference		Reference		Reference	
GA	172 (28.4)	30 (17.4)		1.63 (1.03–2.59)	0.04	1.66 (0.96– 2.87)	0.07	1.74 (0.89–3.42)	0.11
AA	19 (3.1)	5 (26.3)		4.47 (1.75–11.46)	0.002	11.51 (4.16–31.87)	<0.001	9.64 (2.40–38.71)	0.001
**Validation cohort**	401		14.1	1.06 (0.62–1.81)	0.83	1.47 (0.82–2.62)	0.20	1.59 (0.85–2.96)	0.15
GG	296 (73.8)	45 (15.2)		Reference		Reference		Reference	
GA	97 (24.2)	12 (12.4)		0.88 (0.46–1.66)	0.69	1.11 (0.57–2.13)	0.76	1.15 (0.57–2.32)	0.7
AA	8 (2)	2 (25)		2.36 (0.57–9.78)	0.24	13.68 (2.91–64.41)	0.001	15.91 (3.27–77.34)	0.001
**Combined Cohorts^a^**	1,007		16	1.51 (1.12–2.04)	0.007	1.55 (1.11–2.17)	0.01	1.52 (1.03–2.23)	0.03
GG	711 (70.6)	93 (13.1)		Reference		Reference		Reference	
GA	269 (26.7)	42 (15.6)		1.25 (0.68–2.27)	0.14	1.40 (0.92–2.16)	0.48	1.43 (0.89–2.34)	0.46
GA	27 (2.7)	7 (25.9)		3.67 (1.68–8.08)	0.002	12.06 (5.16–28.5)	<0.001	12.06 (4.22–34.12)	<0.001
**rs7851696 (G>T)**									
**Discovery cohort**	606		17.9	1.53 (1.06–2.19)	0.02	1.05 (0.71–1.57)	0.80	0.99 (0.58–1.72)	0.99
GG	409 (67.5)	48 (11.7)		Reference		Reference		Reference	
GT	177 (29.2)	32 (18.1)		1.76 (1.12–2.77)	0.01	1.52 (0.92–2.53)	0.11	1.57 (0.78–3.17)	0.21
GT	20 (3.3)	3 (15.0)		1.56 (0.48–5.02)	0.46	0.48 (0.13–1.74)	0.27	0.23 (0.03–2.12)	0.20
**Validation cohort**	401		17	1.09 (0.67–1.77)	0.73	1.26 (0.77–2.06)	0.37	1.22 (0.74–2.02)	0.44
GG	274 (68.3)	41 (15)		Reference		Reference		Reference	
GT	118 (29.4)	15 (12.7)		0.87 (0.48–1.57)	0.64	1.00 (0.55–1.85)	0.99	0.97 (0.52– 1.81)	0.92
GT	9 (2.2)	3 (33.3)		2.35 (0.73–7.61)	0.15	3.07 (0.89–10.56)	0.08	2.86 (0.81–10.09)	0.10
**Combined Cohorts^a^**	1,007		17.5	1.33 (0.99–1.78)	0.05	1.16 (0.87–1.54)	0.33	1.16 (0.83–1.63)	0.40
GG	683 (67.8)	89 (13)		Reference		Reference		Reference	
GT	295 (29.3)	47 (15.9)		1.27 (0.64–2.51)	0.13	1.28 (0.85–1.92)	0.14	1.20 (0.75–1.92)	0.21
GT	29 (2.9)	6 (20.7)		1.90 (0.83–4.39)	0.15	1.22 (0.20–7.46)	0.95	0.97 (0.09–11.02)	0.89

*MAF, minor allele frequency*.

a *The Random effect model of two cohorts combined was analyzed by Meta analysis*.

b *Model 1 is Cox regression analysis before adjustment*.

c *Model 2 is Cox regression analysis adjusted for age, sex, eGFR, SBP, DBP, proteinuria, ACEI/ARB and Glucocorticoid treatments*.

d *Model 3 is Cox regression analysis adjusted for covariates in Model 2 plus Oxford MESTC score*.

### Validation of the Correlation Between Two Variations and IgAN Prognosis in the Validation Cohort

In the validation cohort of 401 patients, the association of the rs1800450-AA genotype with ESRD remained significant after being fully adjusted by clinical and histological variables (HR, 15.91; 95% CI, 3.27–77.34; *P* = 0.001). However, the association between rs7851696 and ESRD failed to be verified in the validation cohort (Table 3).

### Associations Between Two Variations and the Phenotype of IgAN Patients in the Combined Cohorts

Next, the two cohorts were combined by meta-analyses. The results showed that the rs1800450-AA genotype was still an independent risk factor for ESRD (HR, 12.06; 95% CI, 4.22–34.12; *P* < 0.001), while rs7851696 lost significance in the Cox regression models (Table 3).

**Figure 3:**
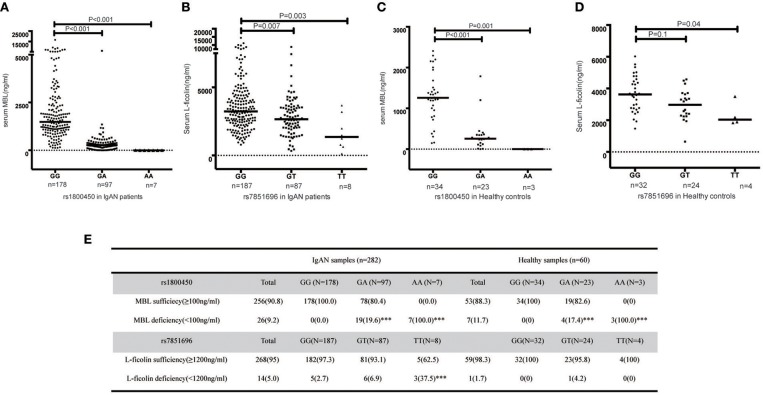
Serum MBL/L-ficolin levels in IgAN Patients and Healthy Controls with different genotypes of rs1800450 or rs7851686. **(A)** MBL levels distributed in rs1800450 of 282 IgAN patients. The median MBL level of the AA group [(1 (0–6) ng/ml, *p* < 0.001] and GA group [(273 (0–6,016) ng/ml, *p* < 0.001] were significantly lower than the GG group [(1,484 (124–16,574) ng/ml]; **(B)** L-ficolin levels distributed in rs7851696 of 282 IgAN patients. The median serum L-ficolin levels of TT [(1,357 (123–3,669) ng/ml, *p* = 0.003] and GT [(2,660 (334–8,807) ng/ml, *p* = 0.007] were lower than GG groups [(3,236 (785–14,417) ng/ml]; **(C)** MBL levels distributed in rs1800450 of 60 healthy control. The median MBL level of the AA group [0 (0–1.2) ng/ml, *P* = 0.001] was also significantly lower than the GG group [1,259 (147–2,409) ng/ml], followed by GA group [255 (1–1786) ng/ml, *P* < 0.001]; **(D)** L-ficolin levels distributed in rs7851696 of 60 healthy control. The median serum levels of L-ficolin were also lowest in TT [2,039 (1,839–3,497) ng/ml, *P* = 0.04), followed by GT [2,974 (650–4,577) ng/ml, *P* = 0.1], while compared to GG [3,631 (1,471–6,025) ng/ml] in 60 healthy controls. **(E)** Serum deficiency of MBL/L-ficolin among three genotype groups of rs1800450 or rs7851696 in 282 IgAN patients and 60 healthy controls. ^***^P < 0.001.

Significant differences in serum MBL levels among the three genotypes in rs1800450 were observed in both patients (median serum MBL levels were 1484, 273, and 1 in IgAN patients with rs1800450-GG, GA and AA, respectively, *p* < 0.001) and healthy controls (median serum MBL levels were 1259, 255, and 0 in healthy controls with rs1800450-GG, GA and AA, respectively, *p* < 0.001). Notably, serum MBL was completely undetectable in 2.5% of IgAN patients and 5% of healthy controls with rs1800450-AA. In addition, the difference in serum L-ficolin levels among the three genotypes in rs7851696 was also significant in IgAN patients (median serum L-ficolin levels were 3236, 2660, and 1357 in IgAN patients with rs7851696-GG, GT and TT, respectively, *p* = 0.001) and healthy controls (median serum L-ficolin levels were 3631, 2974, and 2039 in IgAN patients with rs7851696-GG, GT, and TT, respectively, *p* = 0.021) (Figure 3).

**Figure 4:**
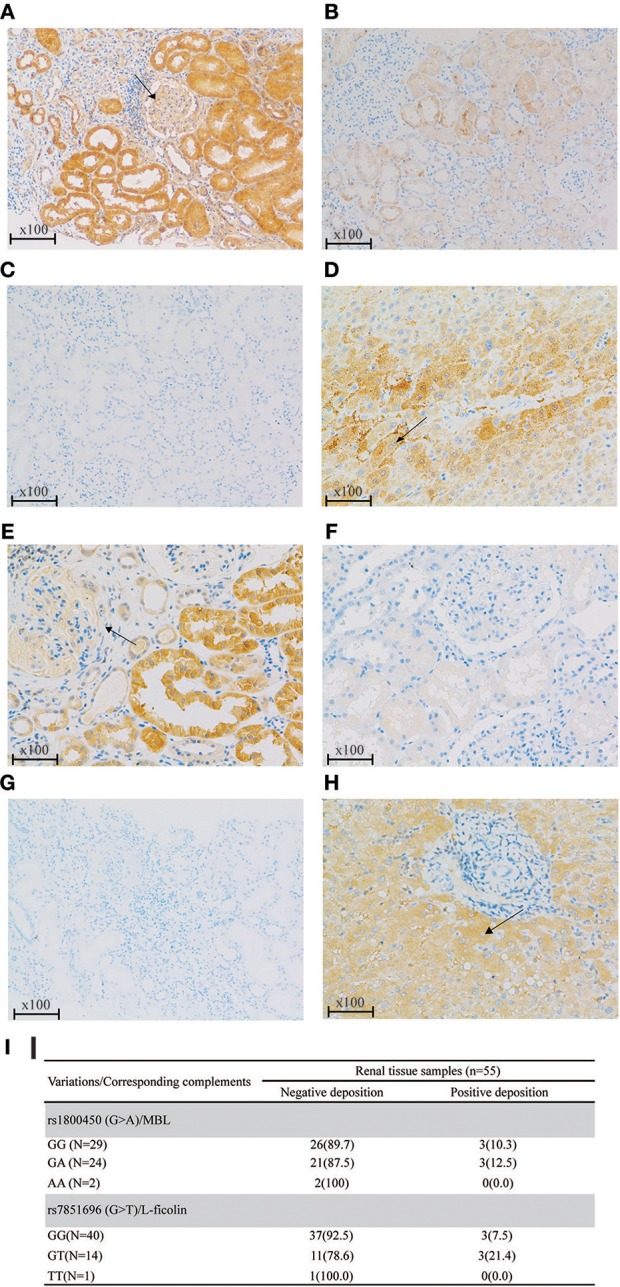
Renal deposition of MBL and L-ficolin (Immunochemical staining, original magnification × 100) among different genotypes of rs1800450 and rs7851696. **(A)** MBL positive deposition in glomerular (arrow) and tubular; **(B)** MBL negative deposition; **(C)** PBS negative control of MBL; **(D)** Liver positive control of MBL in the cytoplasm of hepatocytes (arrow); **(E)** L-ficolin positive deposition in glomerular (arrow) and tubular; **(F)** L-ficolin negative deposition; **(G)** PBS negative control of L-ficolin; (**H**) Liver positive control of L-ficolin in the cytoplasm of hepatocytes (arrow);**(I)** MBL/L-ficolin deposition among different genotypes of rs1800450 and rs7851696 in 55 IgAN patients. PBS, phosphate buffered solution. Renal specimens were from those IgAN patients at their pathological diagnosis. The liver specimen is normal liver tissue next to tumor of a liver cancer patient.

Based on the previous literature, MBL deficiency was defined as a serum MBL level <100 ng/ml (10), and L-ficolin deficiency was defined as a serum L-ficolin level <1,200 ng/ml (29). For rs1800450, the AA group demonstrated the highest proportion of serum MBL deficiency compared with the GA and GG groups. Similar results were also found for rs7851696 (Figure 3). Intriguingly, renal deposition of MBL was negative in both patients with rs1800450-AA, and renal deposition of L-ficolin was also negative in the only one patient with rs7851696-TT, suggesting an inactivation of the MBL pathway in these patients (Figure 4). We only observed the positive deposition in patients with sufficient complement protein level (Supplementary Table 5). By multivariate Cox regression analysis, only MBL deficiency was significantly associated with ESRD (Supplementary Table 6).

We further analyzed the associations between MBL-rs1800450 and baseline clinical and pathological variables, including systolic and diastolic blood pressure, eGFR and urinary protein excretion and Oxford-MESTC. We found that E1 (OR, 0.11; 95% CI, 0.02–0.84; *P* = 0.03) and T2 (OR, 3.38; 95% CI, 1.55–7.34; *P* = 0.002) were associated with an increased risk for ESRD in patients with AA after adjustment for age and sex (Table 4). Additionally, no significant differences were observed in the distribution of baseline clinical indicators and histological grades between the different genotype groups of rs7851696.

**Table 4:** Clinical and pathologic characteristics of all patients with different genotypes of rs1800450.

**Variables**	**GG**	**GA**	**AA**	**GA vs. GG**		**AA vs. GG**	
				**Beta/OR (95%CI)**	***P*-value^a^**	**Beta/OR (95%CI)**	***P*-value^a^**
*N* (%)	618 (71.2)	231 (26.6)	19 (2.2)	–	–	–	–
eGFR, mL/min/1.73 m^2^	81.0 (15.7–163.9)	73.9 (15.4–144.9)	68.8 (17.3–117.5)	−2.66 (−6.69–1.37)	0.2	−0.19 (−5.67–5.3)	0.95
SBP, mm Hg	126.8 ± 16.9	128.2 ± 17.4	130.3 ± 15.6	0.94 (−1.34–3.23)	0.42	1.12 (−1.97–4.21)	0.48
DBP, mm Hg	80.2 ± 12.1	80.4 ± 12.4	81.1 ± 8.6	−0.01 (−1.68–1.66)	0.99	0.32 (−1.92–2.57)	0.78
Urine protein, g/d	1.2 (0.01–15.2)	1.4 (0.1–12.6)	1.0 (0.1–2.9)	0.04 (−0.24–0.32)	0.79	−0.30 (−0.68–0.09)	0.13
M1 (Ref: M0)	237 (38.3)	82 (35.5)	5 (26.3)	0.92 (0.67–1.26)	0.60	0.62 (0.22–1.75)	0.36
E1 (Ref: E0)	211 (34.1)	88 (38.1)	1 (5.3)	1.17 (0.86–1.61)	0.32	0.11 (0.02–0.84)	0.03
S1 (Ref: S0)	474 (76.7)	186 (80.5)	15 (78.9)	1.28 (0.88–1.87)	0.2	1.23 (0.40–3.80)	0.71
T2 (Ref: T0 plus T1)	73 (11.8)	26 (11.3)	5 (26.3)	1.31 (0.91–1.88)	0.15	3.38 (1.55–7.34)	0.002
C2 (Ref: C0 plus C1)	41 (6.6)	13 (5.6)	0 (0)	0.79 (0.41–1.51)	0.48	–	–

*eGFR, estimated glomerular filtration rate; SBP, systolic blood pressure; DBP, diastolic blood pressure; M, mesangial hypercellularity; E, endocapillary proliferation; S, segmental glomerulosclerosis; T, tubular atrophy and interstitial fibrosis; C, crescents; OR, odd ratio; CI, Confidence interval*.

a *Adjusted by age and sex*.

### Subgroup Analyses of rs1800450 in the Combined Cohorts

**Figure 5:**
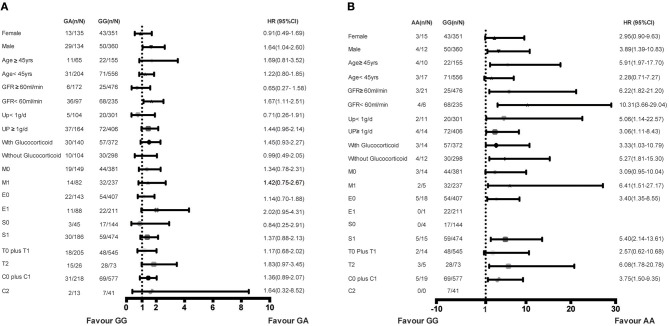
Increased risk of ESRD of genotypes in rs1800450 in different subgroups of combined cohort. **(A)** GA vs. GG genotypes in subgroups of gender, Age, GFR, 24 h urine protein, Glucocorticoid treatment and OXFORD-MESTC; **(B)** AA vs. GG genotypes in subgroups of gender, Age, GFR, 24 h urine protein, Glucocorticoid treatment and OXFORD-MESTC; *n*, number of ESRD; *N*, number of the genotype group; HR, Hazard ratio; CI, confidential interval; M, mesangial hypercellularity; E, endocapillary proliferation; S, segmental glomerulosclerosis; T, tubular atrophy and interstitial fibrosis; C, crescents.

Subgroup analysis was performed in the combined cohort to evaluate the association of rs1800450 with ESRD in the different subgroups. Compared with the GG group, the AA genotype group showed an increased risk for ESRD in male patients (HR, 3.89; 95% CI, 1.39–10.83) or in patients aged <45-years (HR, 5.91; 95% CI, 1.97–17.70), with an eGFR <60 ml/min (HR, 10.31; 95% CI, 3.66–29.04), proteinuria <1 g/d (HR, 5.06; 95% CI, 1.14–22.57), M1 (HR, 6.41; 95% CI, 1.51–27.17), T2 (HR, 6.08; 95% CI, 1.78–20.78), and those who were not treated with steroids (HR, 5.27; 95% CI, 1.81–15.30). Additionally, compared with the GG group, the GA genotype group showed an increased risk for ESRD in male patients (HR, 1.64; 95% CI, 1.04–2.60) or in patients with an eGFR <60 ml/min (HR, 1.67; 95% CI, 1.11–2.51) (Figure 5).

**Figure 6:**
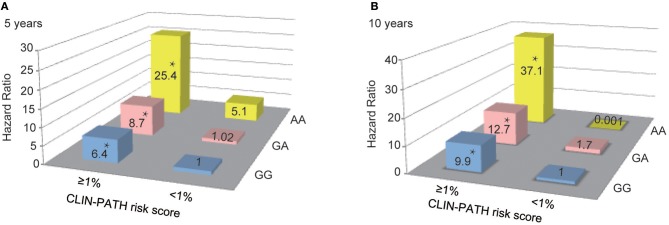
Combined Effect of Baseline CLIN-PATH Risk Score and rs1800450 Genotype in Predicting ESRD at 5-year **(A)** or 10-year **(B)** after Renal Biopsy. ^*^P < 0.05. CLIN-PATH risk score was calculated based on equations from Xie J's study (23). **(A)** Predicting ESRD at 5-years, comparing to GG group with ESRD risk <1%, patients with GA and ESRD risk <1% had a 1.02-fold higher risk (95% CI, 0.28–3.78) of ESRD, patients with AA and ESRD risk <1% had a 5.1-fold higher risk (95% CI, 0.65–40.46) of ESRD, patients with GG and ESRD risk ≥1% had a 6.4-fold higher risk (95% CI, 3.20–12.91) of ESRD; patients with GA and ESRD risk ≥1% had a 8.7-fold higher risk (95% CI, 4.14–18.40) of ESRD; patients with AA and ESRD risk ≥1% had a 25.4-fold higher risk (95% CI, 7.76–83.01) of ESRD; **(B)** Predicting ESRD at 10 years, comparing to GG group with ESRD risk <1%, patients with GA and ESRD risk <1% had a 1.7-fold higher risk (95% CI, 0.15–18.49) of ESRD, patients with AA and ESRD risk <1% had a 0.001-fold higher risk of ESRD, patients with GG and ESRD risk ≥1% had a 9.9-fold higher risk (95% CI, 2.42–40.24) of ESRD; patients with GA and ESRD risk ≥1% had a 12.7-fold higher risk (95% CI, 3.05–53.11) of ESRD; patients with AA and ESRD risk ≥1% had a 37.1-fold higher risk (95% CI, 7.17–192.13) of ESRD.

Finally, the combined effects of rs1800450 and the CLIN-PATH equation, a previously reported ESRD risk calculation tool based on clinical and pathological variables, on the risk prediction for ESRD were investigated (Figure 6). We found that in comparison to patients with GG and ESRD risk <1%, patients with either AA or ESRD risk ≥1% had a 5.1 ~ 8.7-fold higher risk (95% CI, 0.65–40.46) of ESRD after 5-years, while patients with both the AA genotype and ESRD risk ≥1% had a 25.4-fold higher risk (95% CI, 7.76–83.01) of ESRD after 5 years. Similarly, patients with both rs1800450-AA and ESRD risk ≥1% had a strikingly increased ESRD risk after 10 years by 37.1-fold (95% CI, 7.17–192.13).

## Discussion

To date, more than 20 susceptibility loci have been identified based on genome-wide association studies (GWAS) of IgAN (1, 30–34). Recent studies have confirmed that rs11089788 in *MYH9* and rs2856717 in *HLA-DQ/DR* might adversely affect the renal outcome of IgAN (35, 36). Our previous study has demonstrated that a common variation, deletion of complement factor H–related 3,1 genes (CFHR3, 1 Δ), was the causal variant underlying the genome-wide signal of 1q32 and might influence renal interstitial fibrosis in patients with IgAN (19). To date, one of the major challenges of IgAN is still to precisely predict the progression of IgAN, and the identification of novel genetic risk factors involved in disease progression could be useful for achieving this goal.

To our knowledge, this is the first study to systematically investigate the contributions of variations in the lectin pathway to renal function deterioration in IgAN. In the discovery cohort, the *MBL2* and *FCN2* genes were sequenced in 50 patients, resulting in 37 candidate variations. Among them, rs1800450 in *MBL2* and rs7851696 in *FCN2* were separately associated with serum MBL and L-ficolin levels. Both variations increased the risk for ESRD. After validation, only rs1800450 was confirmed as an independent risk factor for ESRD in IgAN. Surprisingly, when combining the two cohorts, rs1800450-AA presented in 2.7% of IgAN patients and obviously increased the ESRD risk by 12.06-fold (95% CI, 4.22–34.12; *P* < 0.001) in comparison to wild-type patients (rs1800450-GG) after adjusting for clinical indicators and the OXFORD-MESTC score. Moreover, the rs1800450-AA genotype was associated with markedly decreased serum MBL levels, renal MBL negative deposition, as well as severe tubulointerstitial damage (OR = 3.38, 95% CI, 1.55–7.34, *P* = 2 × 10^−3^). These results indicated that inactivation of the complement MBL pathway acted independently and adversely affected the renal outcome of IgA nephropathy.

As the most common glomerulonephritis in China, IgAN is an autoimmune disease caused by glomerular deposition of immune complexes (37, 38). The complement system is known to participate in the pathogenesis of IgAN (39). In addition to the alternative pathway, it is generally agreed that complement activation of the lectin pathway (12) plays an important role in the renal injury associated with IgAN (6–9). Deposition of MBL in the renal glomerular mesangial area, co-localizing with IgA, has been found in approximately 20% of IgAN patients (40). Moreover, Roos et al. (9) found that IgAN patients with co-deposition of MBL and L-ficolin had serious renal histological lesions, which indicated the importance of the lectin pathway in IgAN. Additionally, Guo et al. (10) reported that abnormal MBL levels were associated with IgAN progression, especially MBL deficiency. In our study, we validated that IgAN patients with MBL deficiency had an increased risk for disease progression, and we are the first group to report that the haplotype encompassing rs1800450-AA strikingly increased the risk for ESRD in IgAN patients. Ficolin is another important component of the lectin pathway, and the serum concentrations of ficolin clearly correspond to polymorphisms in the *FCN2* gene (17). For rs7851696 in *FCN2*, we did not find any significant association between the investigated variation and clinical/pathological indicators in our study population, except for a trend toward a deleterious effect on ESRD in the univariable Cox model. *FCN2*-rs7851696, however, did not reach statistical significance in the multivariable analysis.

As a C-type lectin secreted by the liver, MBL is a component of the innate immune system due to its opsonizing function, activating macrophages and the lectin pathway (41–43). Serum MBL levels correlate with 6 single nucleotide polymorphisms (SNP) in both exon 1 and the promoter of the *MBL2* gene (44–46). Functionally, these SNPs have been shown to interfere with the formation of MBL oligomers, leading to MBL deficiency and decreased complement activation potential (47). MBL deficiency, defined as an MBL serum concentration below 100 ng/mL, has been estimated to occur in 5–10% of the general white population (48). Similarly, we found that 9.2% of IgAN patients and 11.7% of healthy controls carried the MBL deficiency in our study. Indeed, MBL deficiency influences the susceptibility and the course of different types of infectious and autoimmune diseases (49–52). Until now, most IgAN studies have focused on the associations between renal deposition/serum levels of MBL and severe histological changes/renal outcomes (10, 53, 54). Only a few studies have discussed the genetic basis of MBL deficiency and its association with the prognosis of IgAN.

A report based on an Italian population of 157 IgAN patients and 74 normal controls suggested that the analyzed variations in the *MBL2* gene did not appear to be primarily involved in the susceptibility and even severity of IgAN (55). Furthermore, in 36 Tunisian patients with IgAN and 117 healthy subjects, there was an association between the *MBL2* genotype (rs1800450) and severe sporadic forms of IgAN (56). Among 147 patients with IgAN, Gong et al. (57) found that patients who carried the *MBL2* variant had more immune deposition than wild homozygotes. Shi et al. (58) demonstrated that rs1800450 increased the risk for ESRD in an incomprehensive multivariate Cox model consisting of the initial Scr, DBP, and TID. Overall, these studies of *MBL2* had relatively small sample sizes and short follow-up periods, and they lacked other complement components of the lectin pathway.

In addition, traditional predictors of IgAN progression only explained a small number of variations of renal outcomes in IgAN (59, 60). Efforts have been made to combine clinical and pathological risk factors to increase the predictive performance for IgAN progression (19, 23, 61). A genetic stratification method has also recently been established based on all susceptibility loci identified by GWAS to date (62). However, all these risk models remain to be validated in external cohorts before they can be widely applied in clinical practice. Notably, we found the rs1800450-AA was independent to the most recent clinical risk model, CLIN-PATH risk score. Our patients with both the rs1800450-AA genotype and a high CLIN-PATH score had a 37.1-fold higher 10-year risk (7.17-192.13-fold) of ESRD than patients with GG and a low CLIN-PATH score. Our findings confirm that the rs1800450-AA genotype in the lectin pathway is a novel risk factor for IgAN progression. Lack of serum and renal MBL expression in these patients leads to inactivation of the complement MBL pathway, which may accelerate the progression of IgAN. The underlying mechanism of this process requires further study.

Our study has multiple strengths compared with previous analyses. First, our two-stage design enabled the accurate validation of our genetic predictors. We also use a strict primary endpoint of ESRD in this study, which is more relevant compared with other commonly used endpoints based on eGFR decline or CKD stage. After adjustment for a variety of traditional clinical indicators and treatment, rs1800450 still showed a significant association with a poor renal outcome. There are still several limitations of this study. Due to the retrospective cohort design, longitudinal changes in complement components during the follow-up period were unavailable. In addition, our results must be validated in external cohorts and other populations, since we only included a Chinese population in this study. Finally, whether the contribution of genetic deficiencies in MBL2 to renal disease progression is specific to IgAN and the underling mechanism require further study. Especially, functional studies are needed toconfirm whether IgAN patients carrying rs1800450 had another complement pathways activated outside of the lectin pathway.

## Conclusion

In summary, 2.7% of Chinese IgAN patients carry *MBL2* rs1800450-AA, which is associated with the absence of expression of MBL in both serum and renal tissue. These patients have an obviously elevated risk for renal function deterioration, probably due to inactivation of the complement MBL pathway.

## Ethics Statement

This cohort study was performed in accordance with the Declaration of Helsinki and approved by the Ethics Research Committee from Ruijin Hospital, medical school of Shanghai Jiaotong University. All participants provided written informed consent prior to inclusion in the study.

## Author Contributions

JX and NC conceived and designed the study. JX, HZ and NC supervised the whole study. MS, SY, YJ, ZW, JM, MY, XZ, XP, HR, WW, and LZ collected IgAN cases. YO and MY performed the experiments. YO and JX analyzed the data. YO and JX wrote the paper. Each author contributed important intellectual content during manuscript drafting or revision.

### Conflict of Interest Statement

The authors declare that the research was conducted in the absence of any commercial or financial relationships that could be construed as a potential conflict of interest.
