# Supplementary material for: A Rare Genetic Defect of MBL2 Increased the Risk for Progression of IgA Nephropathy
Source: Front Immunol. 2019 Mar 22;10:537. doi: 10.3389/fimmu.2019.00537 (PMC6438956; doi:10.3389/fimmu.2019.00537)
Supplement: Supplementary file 1 [file Table_1.doc]

**Supplementary Table 1. Primer sequences of *MBL2* and *FCN2* genes**

| Position | Former primer (5'-3') | Reverse primer (5'-3') | Product Length (bp) |
| --- | --- | --- | --- |
| *MBL2* |  |  |  |
| Promoter | ATTCCTGCCAGAAAGTAGAG | GTGATGGAAACAGGGACAT | 731 |
| Exon1 | ACGCAGTGTCACAAGGAATG | GCCAGAGAATGAGAGCTGAA | 509 |
| Exon2 | CTCAGGTGCCCACCATACAG | AGGGCACTGGGTCAGAAAGC | 404 |
| Exon3 | GACATTTGGGGTTGGATGGA | AATGCCTAGCAGGGTACAGA | 371 |
| Exon4 | CCGGCAACTGTACTCTAGAA | GTGATTGCCCACAAAAGGAA | 589 |
| *FCN2* |  |  |  |
| Promoter | CATTGAAGGAAAATCCGATGGG | GTTTGCTAAAGATGTTCTGCTTC | 766 |
| Exon1 | GAGATGATCTCGCACCTCCT | CTGAGATGCCACATCCATTC | 439 |
| Exon2 | TGTCACCAAGATGGCAGATG | AGACGCTCCCTCCATGTCAG | 393 |
| Exon3 | CCTGGATCTAGAACCTTCTG | ACTGAGAACCCACACACACA | 429 |
| Exon4 | TGGCTGGACCCATACCATCA | CATTCTGGCAATGGCGTCGT | 241 |
| Exon5 | CCGCTCTGTTCATACAGACG | CCGACCAGGAGAATAGAGAA | 349 |
| Exon6 | TCCTTCATCCTGTGGAGTCT | GGCCAAGAGGCTCTTGTGTT | 390 |
| Exon7 | CCATGGAGTCCAGACCTCCT | GATGGACGAATTGGCTCAGA | 365 |
| Exon8 | GCCAGGCCTCAGGTATAAA | TACAAACCGTAGGGCCAAGC | 411 |

**Supplementary Table 2. Power calculation**

| MAF | Genotype Hazard Ratio (Log-additive-model) | | | | | | | | | |
| --- | --- | --- | --- | --- | --- | --- | --- | --- | --- | --- |
|  | 1.1 | 1.2 | 1.3 | 1.4 | 1.5 | 1.6 | 1.7 | 1.8 | 1.9 | 2 |
| 0.05 | 8% | 15% | 26% | 41% | 54% | 67% | 78% | **86%** | **92%** | **96%** |
| 0.1 | 10% | 25% | 44% | 67% | **81%** | **91%** | **97%** | **99%** | **100%** | **100%** |
| 0.15 | 12% | 33% | 58% | **82%** | **92%** | **98%** | **100%** | **100%** | **100%** | **100%** |
| 0.2 | 14% | 40% | 68% | **89%** | **97%** | **99%** | **100%** | **100%** | **100%** | **100%** |
| 0.25 | 15% | 45% | 74% | **93%** | **98%** | **100%** | **100%** | **100%** | **100%** | **100%** |
| 0.3 | 16% | 50% | 79% | **96%** | **99%** | **100%** | **100%** | **100%** | **100%** | **100%** |
| 0.35 | 17% | 53% | **82%** | **97%** | **99%** | **100%** | **100%** | **100%** | **100%** | **100%** |
| 0.4 | 18% | 55% | **84%** | **98%** | **100%** | **100%** | **100%** | **100%** | **100%** | **100%** |
| 0.45 | 18% | 56% | **85%** | **98%** | **100%** | **100%** | **100%** | **100%** | **100%** | **100%** |
| 0.5 | 19% | 57% | **86%** | **98%** | **100%** | **100%** | **100%** | **100%** | **100%** | **100%** |

The study power of the minimal detectable effect was at 80% (those power ≥ 80% were bold). MAF, minor allele frequency. The minimum threshold of MAF was defined as 5%, in order to make sure the low-frequency variants also are enrolled in further analysis. Before the following sequence, we estimated that the sample size of 613 cases had enough statistical power (more than 80%) to detect a variant with MAF of 5%, which harzard ratio of ESRD at least 1.8 at significance level of 5%.

***Supplementary Table 3A. Correlation analysis of MBL levels and 16 variations within MBL2 gene in 50 patients***

| Variation | cDNA | Position | MAF | Correlation from References | |
| --- | --- | --- | --- | --- | --- |
|  |  | (AA changes) | (%) | Supported | Not supported |
| rs11003125 | -619C>G | Promoter | 46 | [1], [2], [4] |  |
| rs7100749 | -504G>A | Promoter | 3 |  | [3] |
| rs11003124 | -496A>C | Promoter | 8 |  | [2] |
| rs7084554 | -418A>G | Promoter | 10 |  | [4] |
| rs36014597 | -405A>G | Promoter | 10 |  | [4] |
| rs10556764 | -397_-392del | Promoter | 10 |  | [4] |
| rs7096206 | -290C>G | Promoter | 11 | [2], [4] |  |
| rs189469831 | -258G>A | Promoter | 2 | No publications | |
| rs11003123 | -139C>T | Promoter | 10 |  | [4] |
| rs7095891 | -9-57C>T | 5'UTR | 10 | [4] |  |
| rs1800450 | 161G>A | Exon1 (p. Gly54Asp) | 30 | [1], [2], [4] |  |
| rs4647964 | 187+110G>A | Intron1 | 10 |  | [5] |
| rs1982266 | 188-90T>C | Intron1 | 26 |  | [5] |
| Novel | 305-145A>T | Intron2 | 5 | No publications | |
| rs930508 | 374-28C>G | Intron3 | 20 |  | [5] |
| rs930507 | 378C>G | Exon4 (p. Leu126) | 20 |  | [5] |

UTR, un-translated region; AA, amino acid; MAF, miner allele frequency.

***Supplementary Table 3B. Correlation analysis of L-ficolin levels and 21 variations within FCN2 gene in 50 patients***

| Variation | cDNA | Position | MAF (%) | Correlation from References | |
| --- | --- | --- | --- | --- | --- |
|  |  | (AA changes) |  | Supported | Not supported |
| rs3124952 | -986A>G | Promoter | 7 | [6],[7] |  |
| rs3811143 | -902C>A | Promoter | 13 |  | [7] |
| rs3124953 | -602A>G | Promoter | 2 | [6], [9] | [7] |
| rs373370111 | -564T>C | Promoter | 1 | No publications | |
| rs3811140 | -557A>G | Promoter | 17 |  | [6], [9] |
| rs7865453 | -64A>C | Promoter | 17 |  | [8], [9] |
| rs17514136 | -4A>G | 5'UTR | 5 | [6] | [7] |
| rs7032741 | 100+170C>T | Intron1 | 17 | No publications | |
| rs3124955 | 214+60T>C | Intron2 | 47 |  | [7] |
| rs7024491 | 215-64A>G | Intron2 | 49 |  | [7] |
| rs118122273 | 215-59G>A | Intron2 | 17 | No publications | |
| rs3128624 | 215-9A>G | Intron2 | 47 |  | [7] |
| rs4520243 | 222T>C | Exon3(p.Arg74) | 47 |  | [7] |
| rs7037264 | 268+11G>A | Intron3 | 49 |  | [7] |
| rs148404686 | 301+42C>T | Intron4 | 2 | No publications | |
| rs12684476 | 349G>A | Exon5(p.Gly117Ser) | 2 | No publications | |
| rs34789496 | 543C>T | Exon6(p.His181) | 17 |  | [7] |
| rs12684723 | 559+45G>A | Intron6 | 18 | No publications | |
| rs17549193 | 707C>T | Exon8(p.Thr236Met) | 4 | [10] | [6], [8] |
| rs7851696 | 772G>T | Exon8(p.Ala258Ser) | 18 | [6], [7] |  |
| rs4521835 | *45T>G | Intron8 | 31 |  | [11] |

UTR, un-translated region; AA, amino acid; MAF, miner allele frequency.

**Supplementary Table 4. Frequencies of variations associated with serum MBL or L-ficolin levels and ESRD in discovery cohort (N=606).**

| **Variation (N)** | **MBL/L-ficolin level** | **Gene frequency** | | | **HR (95%CI)** |
| --- | --- | --- | --- | --- | --- |
|  | **(ng/ml)** | **Total** | **ESRD** | **Non-ESRD** |  |
| **MBL2** |  |  |  |  |  |
| **rs7096206 (G/C)** |  |  |  |  |  |
| GG (N=443) | 717.6(0-16574.03) | 0.731 | 0.735 | 0.73 | -reference- |
| CG (N=150) | 976.4(0.00-5209.0) | 0.248 | 0.253 | 0.247 | 2.21(0.30-16.48) |
| CC (N=13) | 304.3(198.7-2720.6) | 0.021 | 0.012 | 0.023 | 1.91(0.26-13.77) |
| **rs7095891 (C/T)** |  |  |  |  |  |
| CC (N=477) | 623.9(0.00-16574.0) | 0.79 | 0.831 | 0.783 | -reference- |
| CT (N=118) | 1261.3(64.0-9186.3)** | 0.195 | 0.169 | 0.2 | 0.81(0.45-1.44) |
| TT (N=9) | 2097.6(740.3-2812.5) | 0.015 | 0 | 0.017 | / |
| **rs1800450 (G/A)** |  |  |  |  |  |
| GG (N=415) | 1368.5(123.9-16574.0) | 0.685 | 0.578 | 0.702 | -reference- |
| GA (N=172) | 323.3(0-6106.2)*** | 0.284 | 0.361 | 0.272 | 1.63(1.03-2.59)* |
| AA (N=19) | 0(0-3.9)*** | 0.031 | 0.06 | 0.027 | 4.47(1.75-11.46)* |
| FCN2 |  |  |  |  |  |
| **rs3124952 (A/G)** |  |  |  |  |  |
| GG (N=3) | / | 0.005 | 0 | 0.006 | / |
| AG (N=80) | 3669.8(1284.0-8806.9) | 0.14 | 0.217 | 0.127 | 1.31(0.77-2.22) |
| AA (N=490) | 3105.1(123.2-14417.4) | 0.855 | 0.783 | 0.867 | -reference- |
| **rs17514136 (A/G)** |  |  |  |  |  |
| CC (N=534) | 2996.8(123.2-14417.4) | 0.883 | 0.831 | 0.891 | -reference- |
| CT (N=71) | 3568.0(1358.5-9739.6) | 0.117 | 0.169 | 0.109 | 1.45(0.81-2.60) |
| TT (N=0) | / | / | / | / | / |
| **rs7851696 (G/T)** |  |  |  |  |  |
| GG (N=409) | 3283.5(785.2-14417.4) | 0.675 | 0.578 | 0.69 | -reference- |
| GT (N=177) | 2638.5(445.8-8806.9) | 0.292 | 0.386 | 0.277 | 1.76(1.12-2.77)* |
| TT (N=20) | 1240.3(123.2-3668.5)* | 0.033 | 0.036 | 0.033 | 1.56(0.48-5.02) |

*, P<0.05; **，P <0.001;***，P <0.001

The complement components levels in different genotypes were compared using the Mann-Whitney *U*-test or the Kruskal-Wallis test.

Analyzing the association between genotypes and end stage of renal disease, univariate cox analysis was performed.

Using the wild type as reference, hemizygote and homozygote groups were separately compared with it.

**Supplementary Table 5. The associations of complement deficiency and renal depositions in 55 patients**

| Serum | Renal deposition | |
| --- | --- | --- |
|  | MBL negative | MBL positive |
| MBL sufficiency (≥100ng/ml) | 39(79.6) | 6(100) |
| MBL deficiency (<100ng/ml) | 10(20.4) | 0(0) |
|  | L-ficolin negative | L-ficolin positive |
| L-ficolin sufficiency (≥1200ng/ml) | 49(100) | 6(100) |
| L-ficolin deficiency (<1200ng/ml) | 0(0) | 0(0) |

**Supplementary Table 6. The associations of two complement deficiencies with ESRD in 282 patients.**

| Serum complement deficiency | ESRD, n (%) | HR (95%CI) | P value |
| --- | --- | --- | --- |
| MBL |  |  |  |
| Sufficiency (n=256) | 24(9.4) | -reference- |  |
| Deficiency (n=26) | 5(19.2) | 3.89(1.17-12.93) | 0.027 |
| L-ficolin |  |  |  |
| Sufficiency (n=268) | 27(10.1) | -reference- |  |
| Insufficiency (n=14) | 2(14.3) | 1.07(0.18-6.55) | 0.94 |

Data were analyzed by cox analysis in 282 patients with serum samples; ESRD, end stage of renal disease; HR, hazard ratio; CI, confidential

ESRD risk is adjusted for estimated glomerular filtration rate, systolic and diastolic blood pressures, 24-hour urine protein, angiotensin-converting enzyme inhibitors and/or angiotensin receptor blockers or glucocorticoid treatments.

Reference:

[1]. [Zhang N](https://www.ncbi.nlm.nih.gov/pubmed/?term=Zhang N%5BAuthor%5D&cauthor=true&cauthor_uid=24376633), [Zhuang M](https://www.ncbi.nlm.nih.gov/pubmed/?term=Zhuang M%5BAuthor%5D&cauthor=true&cauthor_uid=24376633), [Ma A](https://www.ncbi.nlm.nih.gov/pubmed/?term=Ma A%5BAuthor%5D&cauthor=true&cauthor_uid=24376633), [Wang G](https://www.ncbi.nlm.nih.gov/pubmed/?term=Wang G%5BAuthor%5D&cauthor=true&cauthor_uid=24376633), [Cheng P](https://www.ncbi.nlm.nih.gov/pubmed/?term=Cheng P%5BAuthor%5D&cauthor=true&cauthor_uid=24376633), [Yang Y](https://www.ncbi.nlm.nih.gov/pubmed/?term=Yang Y%5BAuthor%5D&cauthor=true&cauthor_uid=24376633), [Wang X](https://www.ncbi.nlm.nih.gov/pubmed/?term=Wang X%5BAuthor%5D&cauthor=true&cauthor_uid=24376633), [Zhang J](https://www.ncbi.nlm.nih.gov/pubmed/?term=Zhang J%5BAuthor%5D&cauthor=true&cauthor_uid=24376633), [Chen X](https://www.ncbi.nlm.nih.gov/pubmed/?term=Chen X%5BAuthor%5D&cauthor=true&cauthor_uid=24376633), [Lu M](https://www.ncbi.nlm.nih.gov/pubmed/?term=Lu M%5BAuthor%5D&cauthor=true&cauthor_uid=24376633).Association of levels of mannose-binding lectin and the MBL2 gene with type 2 diabetes and diabetic nephropathy. PLoS One. 2013 Dec 20;8(12):e83059. (PMID: 24376633)

[2]. Kim MA, Yoon MK, Kim SH, Park HS. Association of MBL With Work-Related Respiratory Symptoms in Bakery Workers. Allergy Asthma Immunol Res. 2017 Jan;9(1):85-91.( PMID: 27826966)

[3]. Zhang DF, Huang XQ, Wang D, Li YY, Yao YG. Genetic variants of complement genes ficolin-2, mannose-binding lectin and complement factor H are associated with leprosy in Han Chinese from Southwest China. Hum Genet. 2013 Jun;132(6):629-40.( PMID: 23423485)

[4]. [Swale A](https://www.ncbi.nlm.nih.gov/pubmed/?term=Swale A%5BAuthor%5D&cauthor=true&cauthor_uid=25170052), [Miyajima F](https://www.ncbi.nlm.nih.gov/pubmed/?term=Miyajima F%5BAuthor%5D&cauthor=true&cauthor_uid=25170052), [Kolamunnage-Dona R](https://www.ncbi.nlm.nih.gov/pubmed/?term=Kolamunnage-Dona R%5BAuthor%5D&cauthor=true&cauthor_uid=25170052), [Roberts P](https://www.ncbi.nlm.nih.gov/pubmed/?term=Roberts P%5BAuthor%5D&cauthor=true&cauthor_uid=25170052), [Little M](https://www.ncbi.nlm.nih.gov/pubmed/?term=Little M%5BAuthor%5D&cauthor=true&cauthor_uid=25170052), [Beeching NJ](https://www.ncbi.nlm.nih.gov/pubmed/?term=Beeching NJ%5BAuthor%5D&cauthor=true&cauthor_uid=25170052), [Beadsworth MB](https://www.ncbi.nlm.nih.gov/pubmed/?term=Beadsworth MB%5BAuthor%5D&cauthor=true&cauthor_uid=25170052), [Liloglou T](https://www.ncbi.nlm.nih.gov/pubmed/?term=Liloglou T%5BAuthor%5D&cauthor=true&cauthor_uid=25170052), [Pirmohamed M](https://www.ncbi.nlm.nih.gov/pubmed/?term=Pirmohamed M%5BAuthor%5D&cauthor=true&cauthor_uid=25170052). Serum Mannose-Binding Lectin Concentration, but Not Genotype, Is Associated With Clostridium difficile Infection Recurrence: A Prospective Cohort Study. Clin Infect Dis. 2014 Nov 15;59(10):1429-36. (PMID: 25170052)

[5]. Kalia N, Sharma A, Kaur M, Kamboj SS, Singh J. A comprehensive in silico analysis of non-synonymous and regulatory SNPs of human MBL2 gene. Springerplus. 2016 Jun 21;5(1):811. (PMID: 27390651)

[6]. Munthe-Fog L, Hummelshøj T, Hansen BE, Koch C, Madsen HO, Skjødt K, Garred P. The Impact of FCN2 Polymorphisms and Haplotypes on the Ficolin-2 Serum Levels. Scand J Immunol. 2007 Apr;65(4):383-92.( PMID: 17386030)

[7]. Hoang TV, Toan NL, Song le H, Ouf EA, Bock CT, Kremsner PG, Kun JF, Velavan TP. Ficolin-2 Levels and FCN2 Haplotypes Influence Hepatitis B Infection Outcome in Vietnamese Patients. PLoS One. 2011;6(11):e28113.( PMID: 22140517)

[8]. Szala A, St Swierzko A, Cedzynski M. Cost-effective procedures for genotyping of human FCN2 gene single nucleotide polymorphisms. Immunogenetics. 2013 Jun;65(6):439-46. (PMID: 23525825)

[9]. Hummelshoj T, Munthe-Fog L, Madsen HO, Fujita T, Matsushita M, Garred P.Polymorphisms in the FCN2 gene determine serum variation and function of Ficolin-2. Hum Mol Genet. 2005 Jun 15;14(12):1651-8. (PMID: 15879437 )

[10]. Mishra A, Antony JS, Sundaravadivel P, Tong HV, Meyer CG, Jalli RD, Velavan TP, Thangaraj K. Association of Ficolin-2 Serum Levels and FCN2 Genetic Variants with Indian Visceral Leishmaniasis. PLoS One. 2015 May 12;10(5):e0125940.( PMID: 25965808)

[11]. Dabrowska-Zamojcin E, Czerewaty M, Malinowski D, Tarnowski M, Słuczanowska-Głabowska S, Domanski L, Safranow K, Pawlik A.Ficolin-2 Gene rs7851696 Polymorphism is Associated with Delayed Graft Function and Acute Rejection in Kidney Allograft Recipients. Arch Immunol Ther Exp (Warsz). 2018 Feb;66(1):65-72.( PMID: 28536887)
